# Addressing the challenges and relational aspects of index-linked HIV testing for children and adolescents: insights from the B-GAP study in Zimbabwe

**DOI:** 10.1186/s43058-020-00091-9

**Published:** 2020-11-04

**Authors:** Chido Dziva Chikwari, Sarah Bernays, Stefanie Dringus, Victoria Simms, Helen A. Weiss, Edwin Sibanda, Katharina Kranzer, Gertrude Ncube, Rudo Chikodzore, Karen Webb, Trevor Chirimambowa, Kenny Sithole, Nonhlanhla Ndondo, Tsitsi Apollo, Miriam Mutseta, Rashida A. Ferrand

**Affiliations:** 1 Clinical Research Department, London School of Hygiene and Tropical Medicine, London, UK; 2 Biomedical Research and Training Institute, 10 Seagrave Road, Avondale, Harare, Zimbabwe; 3 Global Health Department, London School of Hygiene and Tropical Medicine, London, UK; 4 School of Public Health, University of Sydney, Sydney, Australia; 5 MRC Tropical Epidemiology Group, London School of Hygiene and Tropical Medicine, London, UK; 6 Health Services Department, Bulawayo, Zimbabwe; 7 Ministry of Health and Child Care, Harare, Zimbabwe; 8 Organization for Public Health Interventions and Development, Harare, Zimbabwe; 9 Million Memory Project Zimbabwe, Bulawayo, Zimbabwe; 10 Population Services International, Harare, Zimbabwe

**Keywords:** HIV testing, Index-linked testing, Barriers, Children, Adolescents

## Abstract

**Introduction:**

Index-linked HIV testing, targeted at sexual contacts or children of individuals with HIV, may improve yield and efficiency. The B-GAP study evaluated index-linked testing approaches in health facility and community-based settings. This paper reports on a qualitative study to understand factors that affect uptake of index-linked HIV testing for children and adolescents.

**Methods:**

We conducted four focus group discussions (FGDs) with caregivers who had their children tested through B-GAP and one FGD with providers who offered index-linked HIV testing to indexes. We aimed to understand enabling and inhibiting factors in the decision-making process. Translated and transcribed transcripts were read for familiarisation. Following initial coding, analytical memos were written to identify emerging key themes across the data.

**Results:**

Our findings showed there was inadequate emphasis on paediatric HIV in routine care which had a negative impact on subsequent uptake of testing for children. Once the decision to test had been made, access to facilities was sometimes challenging and alleviated by community-based testing. A key finding was that HIV testing is not a discrete event but a process that was influenced by relationships with other family members and children themselves. These relationships raised complex issues that could prevent or delay the testing process.

**Conclusion:**

There is a need to improve messaging on the importance of HIV testing for children and adolescents and to provide support to caregivers and their families in order to improve testing uptake. Addressing access barriers through the provision of community-based testing and implementing a family-centred approach can optimise index-linked testing.

**Supplementary Information:**

The online version contains supplementary material available at 10.1186/s43058-020-00091-9.

Contributions to the literature

- Although promoted and recommended by the World Health Organization, index-linked HIV testing for children has not been a standard practice in routine HIV care for many countries including Zimbabwe.
- Prior this study, no study has evaluated the factors that influence and affect uptake of index-linked HIV testing for children and adolescents as reported in our manuscript.
- Our findings have the potential to bridge the HIV testing gap for children and optimise index-linked testing. This strategy has been shown to result in higher yield of HIV when compared to universal HIV testing; however, index-linked testing uptake remains suboptimal.

## Introduction

Globally, 2.8 million children aged 0–19 years were living with HIV in 2018 [1, 2]. While coverage of prevention of mother to child transmission programmes (PMTCT) has increased (82% in 2018 compared to 43% in 2013), corresponding coverage of early infant diagnosis of HIV remains low (59% in 2018) [3]. Therefore, many children living with HIV are only diagnosed in later childhood, with a consequent increased risk of mortality and morbidity [4].

The World Health Organization (WHO) has recommended targeted HIV testing strategies such as index-linked HIV testing to improve efficiency and reduce costs of HIV testing [5]. Index-linked HIV testing (i.e. HIV testing offered to children or sexual contacts of individuals living with HIV) is anticipated to have higher uptake and yield compared to universal HIV testing approaches. When implemented in Malawi, Kenya, Lesotho and Cameroon, index-linked testing for children did result in a higher yield of HIV (proportion of eligible children who test positive) compared to universal testing, but uptake of testing (proportion of eligible children tested) remained suboptimal, ranging from 14 to 71% [6–8].

Children and adolescents face specific barriers to accessing HIV testing in facilities, including the requirement for parental consent, perceived low risk in this age group by healthcare providers who then do not offer testing, and lack of personal resources to independently access HIV testing [9]. Index-linked HIV testing may mitigate some of these barriers by targeting and follow up of children and adolescents at risk of HIV. However, index-linked testing initially requires uptake by a parent or caregiver, and there are a number of factors that influence the decision-making process by individuals when considering HIV testing for their children. HIV remains a deeply stigmatised infection and therefore a diagnosis of HIV is associated with risk of social harms [10]. HIV infection also requires lifelong treatment which may have a significant impact on children as well as their caregivers’ lives [11]. Therefore, when implementing index-linked HIV testing, it is important to understand the lived experiences of indexes which will influence their ability to engage with HIV testing and care services [12].

The Bridging the Gap in HIV testing and care for children in Zimbabwe (B-GAP) study evaluated uptake and yield of index-linked HIV testing for children and adolescents aged 2–18 years in rural and urban communities in Zimbabwe [13]. Testing in facility and community-based settings was offered to children of individuals living with HIV already accessing treatment. In this paper, we report on the lived experiences of caregivers who went through index-linked HIV testing for children in their households and providers who offered index-linked testing to the caregivers in order to further understand the decision-making process for testing. We aimed to understand enabling and inhibiting factors for testing, which are critical to inform how this testing strategy should be implemented to optimise uptake.

## Methods

### Study setting

Zimbabwe has experienced an early onset, severe and sustained HIV epidemic with antenatal HIV prevalence peaking at 35% in 1998 [14] and current adult HIV prevalence of 14% [2]. In 2016, an estimated 24% of households had at least one HIV-positive household member, and HIV prevalence among children aged 0–14 years in Zimbabwe was 2% [1, 2]; 39% were undiagnosed and only 26% of children born to mothers with HIV received an HIV test within 1 year of birth [2].

**Fig. 1:**
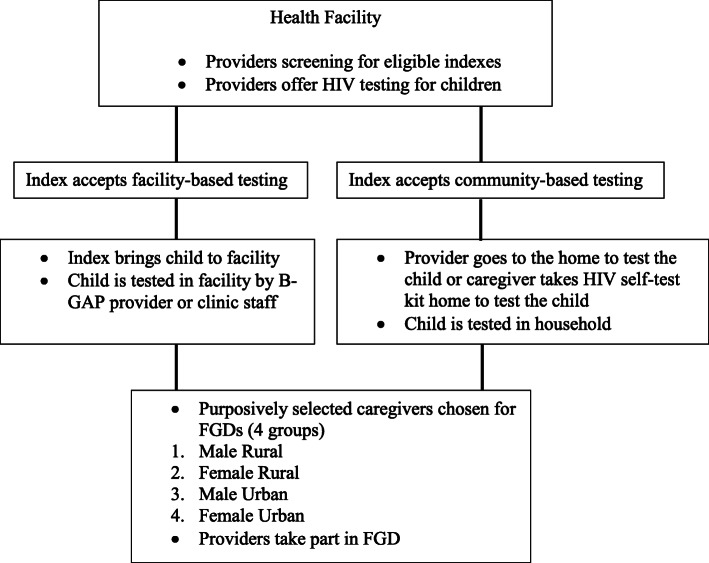
Participant recruitment flow

The B-GAP study was conducted in three rural and six urban facilities from January to December 2018. Individuals attending for HIV care who had children aged 2–18 years of unknown HIV status in their households (indexes) were offered three options for having their children tested for HIV: health facility-based testing, community-based testing by a provider or provision of an oral HIV test kit to the index to test their child at a location of their choice (Fig. 1). Indexes were followed up by telephone or home visits at specified intervals over 21 days to ascertain test outcomes.

### Qualitative study procedures

We conducted one focus group discussion (FGD) with providers who offered index-linked HIV testing to indexes in the health facility, and four FGDs with indexes who had their children tested for HIV through B-GAP (caregivers). No qualitative data collection was conducted with indexes who did not take up HIV testing for their children due to anticipated difficulties in engaging this group. FGDs with caregivers were conducted up to 4 months after being approached to have their child(ren) tested. The four caregiver FGDs were grouped according to gender and site (Figure 1).

Caregivers were purposively selected from the study sites to represent a mix of those who took up the different test location options. Open invitations were made via telephone call to selected B-GAP participants, and those who were available on the scheduled day were recruited. All the B-GAP providers participated in the provider FGD. All of the caregiver FGDs were conducted in a private room at the health facility and the provider FGD was conducted at the study office. They were facilitated by two Zimbabwean research assistants (one male and one female) who were not involved in the recruitment of caregivers for index-linked testing, in order to minimise the risk of interviewer bias. The researchers (NN and KS) were experienced in qualitative research and had at least Bachelors level education. They both received a 1-day refresher training in qualitative data collection prior to conducting the FGDs. Neither NN nor KS had prior relationships with the participants and introduced themselves and the purpose of the research.

Caregivers selected for the FGDs were purposively selected to represent an equal mix of caregivers who had taken up each of the three testing approaches offered through B-GAP. Facilitators used topic guides (Additional files 1 and 2) to generate discussion focused on understanding the caregiver’s experiences of testing for their children, their preferences for how and where index-linked testing should be conducted as well as to gain insights from both the caregivers and the providers about the enabling and inhibiting factors in the decision-making process around index-linked testing. Given the specificity of the sample inclusion criteria, the topic guides were not piloted but in line with best qualitative practice, iterative data collection and analyses allowed for the guides to be refined after each FG D[15]. All FGDs were face-to face and were conducted in either English or one of the local languages (Ndebele and Shona) depending on the participants preferences. Some participants used the local languages interchangeably as is common practice in this setting. NN and KS took field notes during each FGD.

### Data analysis

FGDs were audio recorded and translated from Shona and Ndebele and transcribed into English by NN and KS. Translation was a discursive process to allow for the identification of correct English words for vernacular terms or to decide on words with equivalent meanings where this was not obvious. The translators provided interview summaries to CDC and were involved in discussing the content of the interviews. Transcripts were read by CDC for familiarisation and open coding. Constant case comparisons were made between the coded caregiver transcripts to identify overarching patterns and differences. Caregiver and provider FGD transcripts were analysed separately then compared for similarity and contrast. Following initial coding, analytical memos were written to identify emerging key themes. Content thematic analysis was done collaboratively by the first two authors (CDC and SB), and recurring themes were noted in the later FGDs [15–17]. The coded data was organised using Microsoft Word and Excel. Refined coding was undertaken to further develop thematic areas and to explore case comparisons across the dataset, including considering the learning from any deviant cases.

### Ethics

Ethical approval was obtained from the Medical Research Council of Zimbabwe, the Institutional Review Board of the Biomedical Research and Training Institute, and the London School of Hygiene and Tropical Medicine ethics committee. Written informed consent was obtained from all study participants (caregivers and providers). As part of the consent process and prior to commencing the FGD, participants were made aware that their HIV status would be implicitly disclosed to others in the group and that they could withdraw participation at any point. Participants were not incentivised for participation but reimbursed transport costs. Due to confidentiality concerns surrounding deductive disclosure in the home, FGD transcripts were not given to participants. A dissemination meeting with participants is scheduled at the end of the B-GAP study.

## Results

We conducted five FGDs with 30 caregivers and seven providers between December 2018 and February 2019. The number of participants in each caregiver FGD ranged from 4 to 11. The caregiver FGDs lasted over 2 h on average, and the provider FGD was conducted over 5 h in two sessions. The median age of caregivers was 37 years (IQR 32–45) and 21 (70%) were female (Table 1). Most caregivers (68%) interviewed were biological parents of the children tested. In the main B-GAP study (89%) of indexes offered testing for children in their households had been diagnosed with HIV over a year prior to being offered testing and only 9% had not initiated antiretroviral therapy (ART).

**Table 1:** Participant demographics

**Caregivers** ***N*** **= 30**		
		*n* (%)
**Age category**^**a**^	18–25	2 (6.7)
	25–50	20 (99.7)
	> 50	7 (23.3)
**Sex**	Male	9 (30.0)
	Female	21 (70.0)
**Site**	Rural	15 (50.0)
	Urban	15 (50.0%)
**Test location**	Facility-based	11 (36.7)
	Community-based by provider	15 (50.0)
	Caregiver-provided self-test	4 (13.3)
**Relation to child** ^**b**^ N = 78	Biological parent	53 (67.9)
	Grandparent	18 (23.1)
	Other relation	5 (6.4)
	Non-relation	2 (2.6)
**Providers** ***N*** **= 7**		
		*n* (%)
**Age category**	18–25	1 (14.3)
	25–50	6 (85.7)
	> 50	0 (0.0)
**Sex**	Male	2 (28.6)
	Female	5 (71.4)
**Site**	Urban	4 (57.1)
	Rural	3 (4.3)

^a^Missing data for 1 caregiver

^b^Caregivers had different relationships to children that were tested in their households

Three key themes were identified that explain caregivers’ uptake of testing and the decision-making process around index-linked testing: (i) inadequate emphasis on paediatric HIV information in routine adult care, (ii) the relational nature of index-linked HIV testing of children and (iii) limited access to facilities once the decision to test has been made. Additional provider perspectives that were unique to those voiced by the caregivers included logistical challenges in locating homes for community testing, follow-up and indirect refusals through the provision of wrong addresses and phone numbers.

### Inadequate emphasis on paediatric HIV in routine adult care

Caregivers felt confident about understanding the implications of their own HIV infection, but not how HIV infection would affect their children. Although they had children in their care, for some caregivers, literacy about paediatric HIV was poor, particularly understanding of perinatal HIV and testing for children. This information was not given much emphasis in their routine care, and some caregivers only became meaningfully aware of the need for testing children and the possibility and benefits of testing through their involvement in the B-GAP study. Caregivers reported that the way information was provided in B-GAP enabled them to understand the need to test.

We had only seen posters about free testing and if you are infected, you start on your medication. However, we had not heard about taking your children for testing, we heard this for the first time from B-GAP. (Caregiver #26, Male, 54)

Participants reported that while information about paediatric HIV testing in routine care is an important first step in encouraging the testing of children, information alone is insufficient. A critical step to encourage caregivers to take up testing was being able to apply the pertinence of this information to one’s personal situation. This required more nuanced and individualised information that would enable caregivers to accurately assess their child’s risk.

With younger children, the ones that you are talking about, maybe those who are 7 and below, their parents were a bit hesitant because they would say; ‘Where would they have gotten the HIV from?’ (Provider#1, Female, 30)

Without adequately tailored information, testing is often not taken up, with delays justified by an incorrect assumption that it is unnecessary. In such situations, testing tends to be done reactively only once illness has developed.

As for me, I had never really heard it as such, that children can get tested. But…. my child was constantly sick. So, I thought to myself that it would be best if I brought in my child for testing. (Caregiver #13, Female, 31)

As such, the provision of tailored information provided within the B-GAP intervention was influential in encouraging the uptake of testing. Many caregivers noted that the B-GAP providers explaining the rationale for testing reassured them and resulted in the caregivers having courage to take up testing. Caregivers valued the time that the providers took to convey information and to help them apply it to their individual context. Caregivers emphasised the benefits of allowing time to discuss and listen to their concerns. For some, these conversations extended over more than one encounter.

I would say that the B-GAP team were an experienced team, who were well taught about their program because they would approach you in a way that make you feel free. One person will approach you and explain what they are doing in a good way that helps you to open up and tell them your status and the team was well trained and open. (Caregiver #22, Male, 52)

### The relational nature of index-linked HIV testing of children

Participants illuminated the complex relational webs that HIV so perniciously affects. Caregivers viewed HIV testing of children not as a “one-off” event but as a *process* with potential consequences on multiple people and relationships.

They described living with HIV as a lifelong burden which the child experienced from a young age. Index-linked testing would also inadvertently reveal their own status to other family members, and they would have to confront this both with the child and others. Many caregivers who had kept their HIV status a secret from others in their household feared that they might lose the control they had exercised over disclosing their own status.

I still remember, there was this case uh, of this particular lady who said; ‘Living with HIV is already a burden to me. It’s quite difficult. So, if you come into my home and test these children, and they come out positive, the assumption will be that I am the one who infected them and that will certainly disturb my family; the makeup of my own particular family’. (Provider #4, Male, 34)

Other reported concerns that would follow a positive HIV test were having to support the medication-taking throughout childhood and disruption of care for other children. Caregivers were living with HIV themselves and for many the potential additional responsibility involved in managing their child’s HIV care alongside their own was undesirable, but testing would at least facilitate access to care. Often caregivers would take time to make this decision and reach a point where they are willing and ready.

This one lady; she had initially refused, then I had shown her my office because I usually screen them where she was. So, she came in later, she said; ‘I realised that in as much as I do not want my child to be tested, but I am already on ART, and it is really helping me. I have seen other people die when they are not taking ART. So… I would rather have my child tested so that we know the way forward than to stay in the oblivion age where he/she doesn’t know what is going on’. (Provider#6, Female, 24)

Caregivers stated that these factors would impede uptake of HIV testing for children but, importantly, they emphasised that they needed both time and support to work through these concerns and reported that feeling under pressure to quickly move to testing was not conducive to uptake.

The relational dynamics between caregivers were an important determinant of uptake of index-linked HIV testing for children. Caregivers demonstrated that index-linked HIV testing for children requires negotiation with, and the involvement of, different individuals. In some instances, the decision to test had to be a joint decision between two caregivers such as biological parents. In the B-GAP study, most indexes were women, who sometimes sought the permission of their husbands for testing of their children. Beyond the relational nature of the decision, there are extensive ramifications that could follow a positive HIV test result, including who is deemed responsible for HIV transmission as well as for supporting treatment.

Where the father lives in South Africa, those mothers may not accept the idea of testing the child on their own in the absence of the father of the children because when he returns, he may want answers about where the child got HIV. (Caregiver #11, Female, 37)

While indexes may have been the primary caregiver, they were not always the biological parent of the children living in their households. Caregivers, who were not the biological parents, expressed concern about their right to link a child to testing and care and the negative impact that HIV testing of the child would have on their relations with the biological parents.

If my brother receives a call from me telling him that his child got tested and is now on ARVs, the next time we meet he will certainly kill you. (Caregiver #23, Male, 58)

Regardless of relation to the child, the issue of bearing the additional emotional and financial cost of supporting someone who was not their biological child was raised. However, while acknowledging these issues, many caregivers felt they were liable to make the final decision because they provided day to day care of children. There was variation in how caregivers handled the decision-making, a process which would likely take time to resolve sometimes together with biological parents.

If you are saying that if the children are under your care you would first need to inform a parent in South Africa, what if the child gets sick and needs to be admitted in hospital? What would you do then? In my view, whether the biological parent wants or not I will get the child tested! (Caregiver #19, Female, 42)

I live with my grandchildren; my child lives in South Africa. If anything happens to those children, I am responsible. But I am expected to first inform my child, and if she refuses… I would rather tell her after the fact that I did this and that. (Caregiver #14, Female, 53)

Sometimes we have to take a stand and make the effort to have an open discussion with the biological parents so that it does not become a problem in future. Because it is also a burden to have this opportunity and yet decline to have those non-biological children tested. It is also difficult. So, I need to discuss with the biological parents and inform them of their children’s statuses. I need to be open about it. (Caregiver #10, Female, 65)

In addition to needing to inform biological parents about testing for their children, caregivers who were not the biological parents of the children to be tested noted that the inadvertent disclosure of the HIV status of biological parents was a critical factor to consider in the testing process for non-biological children. Caregivers had to weigh up the importance of testing the child versus the unwelcome risk of doing so potentially revealing the mother’s HIV status, which she may not have wanted to share.

I feared that if the child who isn’t mine biologically tested HIV positive, I was going to struggle to inform the child’s real mother. Perhaps the mother is positive, and she has not disclosed this to me. Initially it used to bother me, but I resolved that I would tell her since she is my younger sister and I tested the child because the child lives under my care. (Caregiver #11, Female, 37)

Caregivers highlighted the need to engage children themselves in the HIV testing process, particularly older children who were often reluctant to go to the clinic. In these cases, caregivers expressed a preference for community health workers to go to the household and speak to older children directly about HIV testing.

This option (home based testing) was very helpful to me because I have an older child who always used to refuse to go with me to the clinic, every time I would ask him to go and get tested at the clinic he would refuse. However, when they arrived, they summoned him, and he could not refuse. And they spoke to him in their way and before I knew it, they were holding hands and they seemed to really click. (Caregiver #2, Female, 36)

For older adolescents, caregivers repeatedly mentioned the need to consider sexual risk rather than solely the perinatal HIV infection assumed by index-linked HIV testing. Caregivers mentioned the fears that adolescents may have about disclosing their sexual behaviour by ways of an HIV test. Older adolescents were felt to be old enough to consent independently.

It’s because they will be afraid, young people indulge and experiment on a lot of things, then that brings in the fear that leads one to decide not to accept this kind of testing. (Caregiver #30, Male, Age-unknown)

### Addressing limited access: provision of community-based HIV testing options

Once the decision to test has been made caregivers often need to bring their children to a health facility. As has been reported in other studies, this leads to out-of-pocket expenditure and loss of income by taking time off work to make the journey.

I had always wanted my children to get tested but I didn’t have the time to take the children to the clinic. (Caregiver #29, Male, 25)

To address these barriers, the B-GAP study provided two options for testing in the community, which participants noted was helpful in increasing accessibility of testing for their children.

We were happy that B-GAP would come home. Taking the children to the clinic using a taxi will be costly since I had 4 children that needed to be tested, so it meant us filling the whole taxi as a family to the clinic and back home, this was going to be too costly and that money would be money that we can use at home for a month, this is what was good about B-GAP. (Caregiver #28, Male, 39)

### Provider perspectives: follow-up, logistics and indirect refusals

Explicit contributions from providers included challenges surrounding community testing by providers and follow-up for caregivers who chose facility testing. Providers noted that sometimes they had difficulty locating households due to inaccurate or wrong addresses and phone numbers provided by the caregivers.

When you are asking about directions then someone says you turn to the left and in actual fact they mean turn to the right, so until you are now in the road and you call and you ask you said I should turn left and they say yes turn to the left but you realize the actual turn is to the right. (Provider#7, Female, 40)

The providers did highlight that in some instances they felt these could be indirect refusals from caregivers who did not want to have the children in their households tested. While the study protocol was that caregivers be followed up at least 3 times in order to complete testing, some caregivers would provide false phone numbers and fake addresses.

I don’t know now because if the number doesn’t go through at all, at all! And the number doesn’t go through, you try for the whole month. You don’t know if this number is some number that belongs to him, but is no longer in use, you know? (Provider#3, Female, 33)

I don’t think there is anything that could have been done, why I say so is because someone lies and tells you that I stay at this address and they do not stay there. (Provider#1, Female, 30)

Despite this, however, there were some caregivers who appreciated the follow-up from providers to facilitate testing for the children in their households. This follow-up allowed caregivers who were indecisive more time to make the decision to test as well as act as a reminder to caregivers who may have forgotten.

It is very good to follow up because when you sign up with B-GAP people, they will call you, I was called, and they asked when they should come. And I told them I was in the village, when I got back, I never got back to them, but they called me again. And they asked when they should come and I appreciated that because I had forgotten about it, so I think that the follow ups are good. If they had gone stealth, I also would have forgotten about it. So, the follow ups are good, some need scolding. (Caregiver #14, Female, 53)

I think it is a good idea to follow up because at times you just agree to be part of the program because you will in a certain state of mind then you set an appointment but then that fear, on the appointment date you decide not to go to the clinic and the clinic staff sees that someone missed their appointment so I think it is good for them to phone you and it will actually give you the confidence that you are dealing with people who are genuine who remembers to call you for a follow up and at times you would just completed a form so that the person does not continue to bother you and in actual essence you were not serious about it, so if they call you it’s now confirmation that whatever the results these people might actually help since they would have made that follow up. (Caregiver #29, Male, 25)

## Discussion

Our study highlights inadequate emphasis on paediatric HIV for adults living with HIV and accessing routine care. Emphasis on testing children has traditionally been confined to PMTCT in most programs and not part of general adult care [18]. Additionally, in general, standard of care focuses more on individual treatment and partner testing rather than for children [19]. Clients receiving HIV care had not been given adequate information about the need to test children in ways that they could satisfactorily engage with. This results in missed opportunities for diagnosis of children who only present once they develop HIV-associated illness. This can be reversed by directly addressing the issue of low-risk perception [20]. B-GAP staff provided tailored information so that caregivers grasped the pertinence of testing their children, which increased uptake of testing.

While many caregivers may be aware of the benefits of HIV testing, they are confronted with a myriad of issues when offered index-linked HIV testing including the prospect of lifelong treatment for their children and automatic disclosure of the HIV status of the index and that of biological parents (if the index is not the biological parent). These issues may have enormous implications on the relationships within a family unit and beyond.

Other studies have also highlighted the difficulties both caregivers and health care providers face in discussing parent-to-child HIV transmission with children, and this can be a substantial barrier to children being tested for HIV [21–24]. HIV status disclosure and potential social harms, such as gender-based violence, are consistent concerns for HIV testing [25, 26]. Support for disclosure to both other family members and to children, where wanted, should be an integral component of index-linked testing. This is likely to have longer-term benefits in terms of promoting psychological well-being and adherence to treatment should the child test HIV-positive [27].

The multiple relationships which may be affected by an HIV test result substantially influence decision-making about testing of children. If uptake of paediatric HIV testing is to be improved, providers need to recognise and more actively engage with these relational dimensions to HIV testing. Our findings illustrate the centrality of relationships in decision-making, which can potentially impede the uptake of testing for children as well as prolonging the process of testing. However, uptake can be improved if providers recognise and engage with individual concerns regarding the complex and potentially wide-ranging consequences of caregivers having their children tested for HIV.

Testing programmes are often focused on the clinical urgency of testing and on achieving targets such as the UNAIDS 90-90-90 targets, without considering social and relational issues [28]. Notably, there has been much more attention paid to these issues for supporting adherence to treatment in children and adolescents through the provision of social and community-based psychosocial support [29, 30]. We argue that a similar approach must be adopted for improving uptake of HIV testing, and HIV testing must be regarded as a *process* rather than a discrete event [31].

Although recommended by WHO, index-linked testing was not a standard practice in routine HIV care for children in Zimbabwe when B-GAP started. The B-GAP study implemented screening of individuals living with HIV and rigorous follow-up by telephone and home visits for caregivers who initially accepted testing for their children. This facilitated tailored conversations between the caregivers and providers, often within the household, with those who would not otherwise have attended healthcare facilities with their children despite agreeing to test [13]. This follow-up may have given caregivers more time and support to make a decision and subsequently led to increased uptake of testing. We note that some participants only took up testing after follow-up and after having received support from B-GAP providers.

In B-GAP, we found that older adolescents (16–18 years) were less likely to be tested when compared to children aged 2–5 years (under review). As highlighted by this and previous studies, engaging adolescents is challenging and index-linked testing may expose sexual activity of adolescents to their caregivers [32, 33]. As highlighted by caregivers, respecting adolescents’ autonomy and approaching them directly and then their caregivers for consent may be more appropriate.

Our study shows that overcoming barriers to access is vital to increase uptake of HIV testing. In B-GAP, the provision of testing in community settings, such as testing at home by a provider or the provision of an oral HIV self-test kit to the caregiver, mitigated against barriers such as transportation costs and loss of income when caregivers have to take time off work to bring children to health facilities. Offering alternative testing approaches can improve uptake of index-linked testing and this has also been demonstrated in Malawi where community testing had higher uptake than facility-based testing [6]. These community-based strategies should be included as part of routine care if we are to identify hard to reach children and adolescents living with undiagnosed HIV. We did note, however, some logistical barriers with uptake of community testing whereby some caregivers gave inaccurate or false addresses or phone numbers. In some instances, providers felt this was indicative of indirect refusals which would be expected where caregivers are not ready to test children in their households.

A key strength of our study was the provision of index-linked HIV testing by trained and dedicated staff. Health facilities in low-income settings are often understaffed and have overworked personnel who may not have the time to offer the support and detailed communication or information that was offered by providers in this study. The B-GAP study facilities are run by nurses. This highlights the limited ability for the study providers and caregivers who are part of this system to influence longstanding change. However, key learnings from this study can be integrated into large-scale implementation. A limitation of our study is that FGDs were held only with caregivers who took up index-linked testing and providers. While it may have been beneficial to include caregivers who refused or did not take up testing for children in their households, this group is difficult to engage as it may be uncomfortable for individuals to justify not acting on health recommendations. However, exploring the decision-making process of caregivers who took up testing provides an insight into the enablers to testing and the issues indexes struggle with. Providers who offered testing to all caregivers (those that took up testing and those that did not) were able to indirectly provide perspectives on both groups through their experiences.

We did not conduct in-depth interviews in our study. FGDs aim to capture social norms around index-linked HIV testing rather than focus on individual stories. However, as participants in each FGD were aware that everyone had engaged in the intervention, it was common for participants to choose to reflect on their own experiences, as well as to consider more common trends within the community. Both sexes and caregivers from both rural and urban settings were represented to allow for the breadth of contextual diversity to be explored.

## Conclusion

In conclusion, this study provides a novel perspective into the lived experiences of providers and caregivers who have offered and accepted index-linked HIV testing in rural and urban settings in Zimbabwe. It demonstrates that the testing gap in children can be bridged by improving paediatric HIV literacy, recognising the relational aspects of HIV testing that caregivers are confronted with when offered HIV testing for their children, and the need for providing time for caregivers to navigate these aspects.

### Panel of recommendations

- Paediatric HIV literacy should be strengthened as part of standard HIV care.
- Discussions about paediatric HIV testing should be individualised and include discussions on HIV risk in children and the benefits of testing.
- Index-linked HIV testing should be coupled with robust support for indexes.
- Offering community-based follow-up and/or an option for community-based HTC may improve access and uptake.
- A family- and process-centred approach should also be adopted to improve uptake of testing
- Adolescents should be directly engaged, and their autonomy respected for HIV testing.

## Supplementary Information

**Additional file 1:.** Caregiver FGD Topic Guide

**Additional file 2:.** Provider FGD Topic Guide

## Data Availability

The datasets used and tools for this study are available from the corresponding author on reasonable request.
