# Supplementary material for: Addressing the challenges and relational aspects of index-linked HIV testing for children and adolescents: insights from the B-GAP study in Zimbabwe
Source: Implement Sci Commun. 2020 Nov 4;1:99. doi: 10.1186/s43058-020-00091-9 (PMC7640428; doi:10.1186/s43058-020-00091-9)
Supplement: Supplementary file 1 — Additional file 1:. Caregiver FGD Topic Guide [file 43058_2020_91_MOESM1_ESM.docx]

**
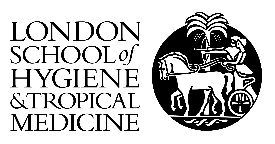
**

**B-GAP**

**Bridging the Gap in HIV Testing and Care for Children in Zimbabwe:**

**Caregiver Focus Group Discussion Guide**

**Introduction [To be read out to all the participants]**

*Thank you for taking the time to speak with me today.*

*My name is _______________ and I am working with the B-GAP project to conduct research on index-linked HIV-testing, treatment and care for children aged 2-18 in Zimbabwe.*

*You all came into contact with a member of the B-GAP team (a research assistant) in your primary care clinic last year. B-GAP was a research study to understand the best ways to provide HIV testing for children living in the households of people living with HIV. B-GAP stands for Bridging the Gap in HIV testing and Care for children in Zimbabwe. In B-GAP index linked testing was used and this is a form of targeted HIV testing where individuals living with HIV were offered HIV testing for children living in their households of unknown HIV status. These individuals were offered three choices for HIV testing for their children; they could bring their child(ren) to the clinic to be tested OR they could choose HIV testing in the community where a research assistant or a community health worker would go to the house to test the child(ren) OR they could choose caregiver testing where the individual could take a HIV self-test kit home to test the child(ren) in their household.*

*Today, I would like to talk to you about your own experiences with index linked HIV-testing through the B-GAP project. I am interested in learning more about how index linked HIV testing can be made more easily accessible, how caregivers perceive index linked testing and the how caregivers can be better supported to access and accept testing for children. I am also interested in learning about your opinions about this approach of HIV testing, why you choose to participate, and why you chose the HIV testing method that you did.*

*The discussion will take about 2 hours. I appreciate you spending this time with me.*

*I am going to audio record the discussion to make sure that I capture all the valuable information that you share with me. I may also write things down while we’re talking so that I don’t forget anything. Participation is voluntary- you do not have to answer any question that you don’t want to, and you can choose to leave the discussion at any time.*

You will be speaking in a group, so you should only share information that you feel is acceptable to discuss in front of others. Before beginning our discussion, we should all agree to keep what is said within this discussion confidential and in the group. As the research team, we will maintain your confidentiality- *We will not record your name anywhere, and no one else will hear the tape of see the notes besides the people who are working on this research project and those in this group. We may use some of what you say in reports or publications but will never use your name.*

*If you have any questions about this study, you can ask me now, or at any time during our conversation* ***(RA: make sure you have collected signed consent form and answered any questions.)***

*Start the* ***tape recorder****.*

**NOTES FOR THE RA:**

1. You are meant to read out the text in green to the participants.
2. Some caregivers would have had several children tested though the different options and it is important that you are aware of this possibility in how you address the group. [tips to be discussed]
3. This topic guide is very dense. To avoid an FDG that is too long please follow the tips below
4. If a topic area has been covered do not go through it again in great detail when you reach the relevant section in the guide instead ask if anyone has anything to add to what has already been said.
5. As a rule of thumb, try to facilitate / manage the group by having one or two participants begin by sharing, and then asking if anyone has something similar / different to add (that way we can see where the agreement / tensions are in the data without everyone inputting.

| **Topic** | **Rationale and Notes** | **Suggested Questions** |
| --- | --- | --- |
| **General discussion** | **INTRO**  *To start off with I would like for us to talk about ourselves, the communities that we live in and the people that we live with in our households. These questions are open for everyone in the group. Please feel free to contribute as we go along.*  ***Rationale:***  *Use this section to get to know the group you are discussing with as well as the communities that they live in. This session should act as an ice breaker getting participants more comfortable discussing in a group and will lay the foundation for the rest of the conversation. It will also allow us to get a better understanding of the participants understanding of index linked testing general understanding of previous testing history of the children in the households of the participants.*  ****** It is important, though, that this part of the discussion doesn’t take up too much time, and prevent us from getting to the other parts of the topic guide*** | 1. We all come from different communities, can you tell me about the different areas that you live in?   [Probes]   - 1. What do you think are some of the similarities between these communities?   2. What do you think are some of the differences between these communities?  1. Can you tell me about the clinics that you are all receiving care in?   [Probes]   1. How far away are the clinics from the community that you live in? 2. How long have you been accessing services from these clinics? 3. Before coming into contact with the B-GAP study had you ever heard about index linked HIV testing for children before?   [Probes]   - 1. What did you hear / know about it?  1. Before the B-GAP study, had anyone ever been offered HIV testing for children in your households?   [Probes]   - 1. If yes, what was that experience like?   2. Was the B-GAP experience different from that experience? If yes, how? |
| **Index linked testing** | **INTRO**  *To start off with we are going to begin our discussion by talking about your experiences of what it was like when the research assistant approached you in the clinic and asked you questions about yourself and the children living in your household. We will also talk about general HIV testing for children and what you believe are the barriers and facilitators of testing.*  ***Rationale:***  *In this section you will talk to the participants in more details about index linked testing and their experiences with having a research assistant approach them in the clinic. This is very important as it forms the premise of the entire testing strategy. Be sure to probe about how they felt about this experience and how it could have been done differently or better from their perspectives.*  *We also want to understand more about the barriers to testing for children who may be exposed to HIV and how these barriers can be eliminated in the context of index linked testing.*  *Where necessary please be sure to probe and be led by the participants throughout the discussion.* | 1. When the research assistant first approached you, what did they say about index-linked testing?    1. What were your thoughts about this type of testing?    2. Did you think it was a good or bad idea?    3. Was there anything that you liked about the way that you were approached for this type of testing?    4. Are there any ways you feel that the way you were approached for testing can be improved?   *[Probes: could this be the place and time where screening was conducted or who conducted the screening, or how they described it?]*   1. Many things may factor in when someone is considering whether or not to accept this type of HIV testing for their children:    1. What might some of these things be?    2. What do you think are some of the difficult things when it comes to accepting this kind of testing?   *[Probes: time spent filling forms in the clinic when you want to go home, disclosing to children / other people why the children are being tested for HIV, the children possibly receiving an HIV test etc.]*   - 1. What are some of the attractive things about accepting this kind of testing?   *[Probes: knowledge of the children in your household’s HIV status, comes from the provider]*   1. What do you think could influence whether or not someone accepts this method of testing? 2. Could it differ based on:    - 1. Younger (<7 years) vs older children (> 7years)?      2. Rural vs urban clinics?      3. Biological children vs non-biological children? 3. How do you feel about your decision to accept this testing for your children?    1. Would you change anything about your decision? 4. Generally, what are some of the barriers to HIV testing for children that you, your friends, family or community members might have faced? 5. What are some of the things that help HIV testing for children? 6. Are there any ways you feel that the way you were approached for testing can be improved?   [Probes: could this be the place and time where screening was done, who conducted the screening or how they described it?] |

| **HIV Testing Options**  **INTRO**  *As I mentioned before all individuals who had children of unknown HIV status living in their household and agreed to index-linked testing were offered 3 options for HIV testing. These options were clinic-based testing (they could bring their child to the clinic to be tested), testing in the community or home by the research assistant or a community health worker and testing by the caregiver who was given a self-test kit to test their child at home.*  *Now we will talk about these different options. In some parts we will all have a general discussion about each of the options and then in some parts I will discuss directly with those that took up the specific option.* | | |
| --- | --- | --- |
| **Clinic based testing** | **INTRO**  *To start off with let us talk about clinic-based testing where caregivers opted to bring their children back to the clinic to be tested for HIV.*  ***Rationale:***  *Clinic based care is what is routinely offered in all clinics. It is therefore important that we elicit all the study participants perspectives about testing in facilities. In this section you will start by a general discussion about this type of testing and then later begin to ask more specific questions to those that took up this type of testing. It will be critical for you to try to keep the group engaged and those sessions that are more focused on those that took up this testing shorter.*  *Please note we are using these interviews to obtain information that will be helpful for us when having focus group discussions with the more specific groups.* | 1. Thinking **in general** about bringing your children back to the facility for testing:    1. What do you feel is attractive /are some of the benefits about this option?   *[probes- access to a nurse or counsellor if you have questions, access to getting started on ART immediately, no disclosure to people in the community]*   - 1. What do you feel is not attractive about / some of the challenges of this option?   *[probes – having to come to the clinic with all your children, transport costs, opening hours of the clinic, disclosure of child HIV status to the clinic staff]*   1. To **those that chose facility-based testing**:    1. what were some of the main reasons why you chose this option?    2. What are some of the reasons why you didn’t select any of the other options?    3. Were there any aspects that you really liked / enjoyed about this option?    4. Are there any challenges that you encountered with facility-based testing?       1. Can you suggest any ways that some of these challenges can be lessened?    5. How do you feel now about your choice (i.e. are you happy with it, or do you wish you would have chosen another option?) 2. As part of the study, participants who chose this option but didn’t attend the clinic with their children for testing were followed up via telephone and then via home visit.    1. How do you feel about this?    2. What were your experiences?    3. Are there ways you feel this could have been improved? 3. Can you describe the experience of HIV testing for your children in the clinic?   *[probes: what went well, what didn’t etc. etc.]*   1. To **those that didn’t chose facility-based testing**:    1. what are some of the main reasons why you didn’t choose this option?    2. Have your thoughts about not choosing this option changed?    3. Is there anything that could have been done differently that would have made you choose this option instead? |
| **Community based testing** | **INTRO**  *Now let us discuss more community-based testing. For this option caregiver opted for a community health worker or research assistant to come to their home to test their children for HIV. We will follow a similar structure to how we did with facility-based testing before.*  ***Rationale:***  *Community based testing is not routinely offered in most clinics and in many of the B-GAP clinics this was conducted in high density suburbs which may have brought out issues of confidentiality. In this testing option often, the RA had to call to schedule an appointment with the caregiver, but it meant that the caregiver never had to leave the comfort of their home. These aspects will be critical as one of the aims of B-GAP is to assess the uptake and acceptability of community-based testing strategies for children.* | 1. Thinking **in general** about this type of testing community-based testing:    1. What do you feel is attractive about / some of the benefits this option?    2. What do you feel is not attractive about / some of the challenges of this option? 2. To **those that chose community-based testing**:    1. What were some of the main reasons why you chose this option?    2. What are some of the reasons why you didn’t select any of the other options?   *[probes - privacy in rented/shared space]*   - 1. Were there any aspects that you really liked / enjoyed about this option?   2. Are there any challenges that you encountered with community-based testing?      1. Can you suggest any ways that some of these challenges can be lessened?   3. How do you feel now about your choice (i.e. are you happy with it, or do you wish you would have chosen another option?)   4. Can you describe the experience of having someone come to your home to test the children in your household for HIV?   5. What did you like about / went well with this?   6. What didn’t you like about / could be improved with this?   7. How did others in your household feel about this testing method (if applicable)  1. To **those that didn’t chose community-based testing**:    1. what are some of the main reasons why you didn’t choose this option?    2. Have your thoughts about not choosing this option changed?    3. Is there anything that could have been done differently that would have made you choose this option instead? |
| **Caregiver testing** | **INTRO**  *Finally, we will talk about caregiver testing. Caregiver testing a novel form of testing where in B-GAP caregivers opted to take an HIV self-test kit home to test children living in their households themselves in the comfort of their home. As before we will follow a similar structure to how we did with facility-based testing and community-based testing before.*  ***Rationale:***  *This is a very new type of testing approach and it is important that the group understands the procedures of this test (a caregiver takes a test kit home to test their child at home).*  *It is important to note that an accuracy assessment may have been conducted for some of these caregivers where a research assistant went with the caregiver to their home and observed them conducting the test on their child. In all settings the caregiver must have attempted to do the test on their own and may have been assisted by the RA.*  *Also note that caregiver testing is conducted using an oral HIV test not a blood-based test. This makes it different from all the other options were children had to have blood drawn.* | 1. Thinking **in general** about caregiver testing: 2. What do you feel is attractive/beneficial about caregiver testing?   [probes: testing in the comfort of your home, don’t have to bring children to the clinic, don’t have to disclose the results to anyone, use an oral test so you don’t have to prick the child]   1. What do you feel is not attractive/ challenging about this option?   [probes: having to perform the test by yourself with no one present to assist, disclosing the test result to your child, getting a positive diagnosis]   1. To **those that chose caregiver testing**: 2. Can you describe the experience of testing your own child for HIV at home? 3. What was most enjoyable about this process? 4. What was least enjoyable about this process? 5. What are some of the main reasons why you chose this testing option? 6. What are some of the reasons why you didn’t select any of the other options?   [probes: privacy-no one gets to see my results, easy to access – no one comes to my house and I don’t have to go anywhere]   1. What are some of the things that you really liked/enjoyed about this testing option? 2. Are there any challenges that you encountered with caregiver testing? 3. How confident / comfortable did you feel preforming the test on your child 4. Did anyone help you preform the test on your child? 5. How did others in your household / your child feel about this testing method (if applicable) 6. Can you suggest any ways that some of these challenges can be lessened? 7. Overall, how do you feel about your choice? (i.e. are you happy with it or do you wish you could have chosen another option?) 8. To **those that didn’t chose caregiver testing** what are some of the main reasons why you didn’t choose this option?   [probes; uncertainty about how to perform the test, how to disclose the result to the child, how well the test performs when compared to blood-based tests] |
| **Scalability, sustainability and wrap up** | **INTRO**  We are now reaching the end of our discussion and as you know B-GAP was only a research study which will end. In this session we want to discuss what your thoughts are about the future of index linked testing in your communities. You will also have an opportunity to tell me anything in addition to the things we have discussed today.  ***Rationale:***  *In our context B-GAP was implemented as a research study. While index linked testing is part of the national HIV testing guidelines it is not often offered from children and clinics are often heavily understaffed and do not offer the service to patients routinely. Based on knowledge of this (discussed in section 1) and experiences of index linked testing we would like to use this section to understand from a client perspective how sustainable this testing method it (including the three options that were available through B-GAP)* | - - - 1. Should index-linked testing continue to be offered for children?   [Probes]   1. Do you think this is something that can be integrated into routine care at the clinic? If yes, why? If no, why?    - 1. What should be kept the same as the way it’s done in B-GAP?      2. What should be done differently to the way it’s done in B-GAP? 2. What are some things that would be needed to make this type of testing be able to be done in other parts of the country? 3. What do you think are the additional resources? (human resources, costs and equipment) 4. What do you think are the additional costs? 5. In future what are your thoughts about having the three options for HIV testing available?    - - 1. Do you have any additional things you would like to add to the things we have discussed? |
| *Thank you all for taking the time do be part of this discussion with me today. We have now reached the end of our focus group discussion****.*** | | |
