# Supplementary material for: Addressing the challenges and relational aspects of index-linked HIV testing for children and adolescents: insights from the B-GAP study in Zimbabwe
Source: Implement Sci Commun. 2020 Nov 4;1:99. doi: 10.1186/s43058-020-00091-9 (PMC7640428; doi:10.1186/s43058-020-00091-9)
Supplement: Supplementary file 2 — Additional file 2:. Provider FGD Topic Guide [file 43058_2020_91_MOESM2_ESM.docx]

**
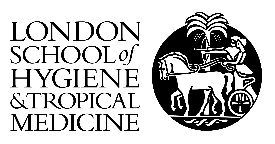
**

**B-GAP**

**Bridging the Gap in HIV Testing and Care for Children in Zimbabwe:**

**Research Assistant Focus Group Discussion Guide**

**Introduction**

Thank you for taking the time to speak with me today.

My name is _______________ and I am working with the B-GAP project to conduct research on index-linked HIV-testing, treatment and care for children aged 2-18 in Zimbabwe.

I would like to talk to you about your own experiences offering HIV-testing through the B-GAP project. I am interested in learning more about how index linked HIV testing can be made more easily accessible, and how caregivers can be better supported to access and accept testing for children. I am also interested in learning about your opinions about this approach of HIV testing.

The discussion will take about 2 hours. I appreciate you spending this time with me.

I am going to audio record the discussion to make sure that I capture all the valuable information that you share with me. I may also write things down while we’re talking so that I don’t forget anything. Participation is voluntary- you do not have to answer any question that you don’t want to, and you can choose to stop the interview at any time.

Everything you say is confidential. We will not record your name anywhere, and no one else will hear the tape of see the notes besides the people who are working on this research project. We may use some of what you say in reports or publications, but will never use your name.

If you have any questions about this study, you can ask me now, or at any time during our conversation ***(RA: make sure you have collected signed consent form and answered any questions.)***

Start the ***tape recorder***.

| **General Topics** | **Rationale and Notes** | **Suggested Questions** |
| --- | --- | --- |
| **Introduction** | Initially we want the group to feel comfortable talking to us, and to provide some information about the communities they have worked in and their work in general.  This is to get the group talking about the work they have been doing and the facilities that they worked in. This will help with framing the rest of the questions in the discussion. Try not to take too long with this section. | 1. Please tell me a little bit about the health facilities that you have worked in? 2. What are there different types of HIV testing services provided in the facilities? 3. Does the facility have any HIV testing services for children and adolescents? 4. Were there any differences you noticed between the different health facilities that you worked in during your time in B-GAP? 5. Please describe your involvement within the facility? 6. What was your role/responsibility? 7. What type of activities did you do within the HIV clinic? |
| **Screening HIV positive participants in clinics and offering Index-linked HIV testing** | We want to learn more about the screening process that the research assistants went through in clinics. We would like to understand some of the challenges that they faced when approaching clients and some of the things they found easiest.  We would also like to understand the reactions of clients in clinics to the B-GAP project and index linked testing in general i.e. when they were offered HIV testing for children in their households and when they were asked questions about themselves and the children in their households. | 1. What were some of the most frequent responses/reactions that you received from clinic patients during screening in clinics? 2. What were some of the encouraging/positive responses received from participants? 3. What were dome of the discouraging/negative responses received from participants? 4. Many indexes said they had no children living in their households, you discussed in meeting meetings that these could have been indirect refusals. How far true do you think this is and what could have been done to reduce the number of refusals? 5. Can you describe your experiences offering HIV testing to caregivers of children of unknown HIV status in your clinics? 6. What were some of the most frequent responses/reactions you got from caregivers after offering index linked testing to children in their households? 7. What were some of the main reasons for refusal of HIV testing from caregivers for eligible children in their households? 8. What were some of the main reasons for acceptance of HIV testing from caregivers for eligible children in their household? 9. Can you describe some of the ways you encouraged caregivers to take up HIV testing for their children? 10. Can you describe some of your experiences with offering HIV testing to caregivers of children who otherwise would not have been thought to be at risk of HIV i.e. older children (>7years)? 11. What were your experiences with offering HIV testing to caregivers who were not biological parents to eligible children in their households (how did these differ from biological parents)? 12. When screening did you note any differences in willingness to test between caregivers of older children (>7 years) and younger children (<7 years)? |
| **Facility based HIV testing** | We really want to understand some of the influencers of choice of index linked testing options i.e. why people chose either of the 3 options available as well as the experiences that the research assistants had with each of the three choices.  Facility based testing was chosen the most and it is important for us to understand why this was so as well as the support that RAs received from the clinic staff. | 1. Facility based testing had the highest uptake, why do you think this was so? 2. What were some of the reasons why people chose clinic-based testing? 3. Do you feel the reasons for choosing clinic-based testing differed between Bulawayo and Mangwe? 4. Do you feel the uptake of clinic-based testing differed between caregivers of older (>7yrs) and younger children (<7 years)? 5. What were some of the reason’s participants gave for selecting clinic-based testing? 6. Did these reasons differ between Bulawayo and Mangwe? 7. Did these reasons differ between clinics? 8. Did these reasons differ between caregivers of older (>7years) and younger (<7years) children? 9. Can you describe some of the challenges of facility-based testing? E.g. follow up on patients? 10. Are there any ways you feel facility-based testing could have been improved? 11. Are there any benefits of facility – based testing compared to the other options? 12. Can you describe your experiences with clinic staff who routinely performed HIV testing? 13. Can you describe the ways through which the clinic staff supported you/didn’t support you for facility-based testing? |
| **Community based testing** | We really want to understand some of the influencers of choice of index linked testing options i.e. why people chose either of the 3 options available as well as the experiences that the research assistants had with each of the three choices.  Community-based testing was the second most popular HIV testing method. Initially PSI and MAC were doing the testing but later it was the Research assistants who started doing the testing due to challenges that MAC and PSI were facing. | 1. What were some of the reason’s participants gave for selecting community-based testing? 2. Did these reasons differ between Bulawayo and Mangwe? 3. Did these reasons differ between clinics? 4. Did these reasons differ between caregivers of older (>7years) and younger (<7years) children? 5. Can you describe some of your experiences with going into people’s homes to test? 6. Can you describe the reception you received in people’s homes? 7. How accurate were people’s directions to their homes? i. Did you have any challenges locating people’s homes? Can you describe these? 8. What were some of the challenges you faced with community-based testing? 9. Are there ways you feel that community-based testing could have been made better? 10. Can you describe your experiences working with community partners PSI and MAC? 11. What do you feel were some of MAC and PSIs strengths? 12. What do you feel were some of MAC and PSIs weaknesses? 13. Why do you think there were difficulties with testing for children through MAC and PSI? |
| **Caregiver testing** | We really want to understand some of the influencers of choice of index linked testing options i.e. why people chose either of the 3 options available as well as the experiences that the research assistants had with each of the three choices.  Caregiver testing was the least popular HIV testing method. It is a novel HIV testing strategy so it will be important to elicit the RAs experiences with caregiver testing, the reasons for uptake and non-uptake as well as the participants perspectives of caregiver testing. | 1. Can you describe your experiences with offering study participants caregiver testing? 2. What were some of the reason’s participants gave for selecting caregiver testing? 3. Did these reasons differ between Bulawayo and Mangwe? 4. Did these reasons differ between clinics? 5. Did these reasons differ between caregivers of older (>7years) and younger (<7years) children? 6. Caregivers testing their own children is something very new: 7. Can you describe the health care workers in the facilities reactions to caregivers testing their own children for HIV? 8. Can you describe some of the caregiver’s initial reactions/responses after offering the caregiver testing option? 9. In general, what the caregiver’s perceptions about caregiver testing (those that took up caregiver testing and those that did not)? 10. What were some of the reasons that caregivers gave for refusing caregiver testing for eligible children in their homes? 11. What were some of the reason that caregivers gave for saying yes to caregiver testing for eligible children in their homes? 12. Do you feel there were any differences between the kind of people that said yes to and those that said no to caregiver testing? 13. Do you feel that caregiver testing works better for some groups of individuals or settings rather than others e.g. those that are more educated? Caregivers of older or younger children? Why is this so? 14. For the accuracy and competency assessments: 15. What were some of the things that caregivers had difficulties with? 16. What were the things that caregivers found easiest? 17. Where there any areas in the instructions that were not clear that you had to explain to the caregivers? 18. How do you think messaging for caregiver testing can be improved? 19. After performing caregiver testing on their children what was the feedback you received from caregivers? 20. Did caregiver perceptions of caregiver testing change after they had performed the test on their children themselves? 21. Were experiences of caregiver testing different in rural and urban settings? 22. Were experience of caregiver testing different for testing of older children (>7 years) and younger children (<7 years) 23. Are there any ways you feel caregiver testing can be done differently? Can you describe these? 24. What sort of strategies do you feel can be used to encourage people to take up caregiver testing? |
| **Follow up of Study Participants** | As part of the study procedures research assistants had to follow up on study participants to either test in the community or remind people to come to the facility for testing. As the study had a community testing component and a requirement for follow up participants it is important for us to understand what was easy or difficult about this process. | You often had to follow up study participants:   1. What were some of the main challenges you encountered when following up study participants? 2. Are there ways you feel that follow up of study participants could be improved? 3. Can you describe your experiences with following up participants who opted for facility-based testing but did not come to the clinic? 4. What were some of the strategies that you used to find children in communities? |
| **Contextual factors:**   1. **Rural and Urban dynamics** 2. **Migration** 3. **Community activities** | B-GAP was intentionally carried out in two settings; a rural one and an urban one.  We believe that there were some key differences in these settings that impacted the uptake of index linked testing, the workload on different tasks in each setting and the choice of testing location.  This session will help us understand some of these differences. | 1. Do you feel there any differences between rural and urban clinics/communities that may have had an impact on index linked testing? Can you describe these differences and their impact? 2. Are there ways that you feel index linked testing should be adapted to accommodate these differences? 3. Do you believe that migration to Botswana and South Africa in our study settings may have had an impact on study outcomes? 4. If yes, in what ways do you feel it impacted the study? 5. If not, why do you feel it had no impact? 6. Do you feel there were significant differences between study clinics?   {Probe: For example, Nkulumane clinic vs Pumula clinic.}   1. Were you aware of other HIV testing initiatives for children in the project communities before you went there or while you were working there? 2. Can you describe some of the initiatives that had the highest impact on B-GAP and how? 3. Can you describe your working relationship with partner organizations?   What were some of the positive aspects? What were some of the challenging aspects?   1. Clinic staff? 2. OPHID staff? 3. MAC? 4. MMPZ? 5. PSI? |
| **Routine index linked testing in facilities, sustainability and Research Assistant perspectives on index linked testing** |  | 1. What was it like to be a research assistant working on the B-GAP project?    1. What were some of the things you enjoyed the most?    2. What were some of the most challenging parts?    3. Were there any parts that changed over time? 2. Are there ways you feel you could have been better supported by the project coordinator and the Biomedical Research and Training Institute? 3. Are there ways you feel you could have been better supported by the clinic staff in the facilitates that you worked in? 4. Before you entered clinics, was index linked testing for children being implemented? 5. What are your observations about routinely implemented index linked testing? 6. How was index linked testing through B-GAP perceived by health care workers in the clinics when you started? 7. Did these perceptions change over time? If they did, in what ways did they change? 8. Do you believe that B-GAP had an impact on health care workers in terms of HIV testing for children? Can you describe the ways in which it had an impact? 9. Do you believe that index linked testing for children will be carried on in B-GAP clinics after you leave? 10. What would need to be done in order for it to be carried on? 11. What are your personal thoughts about index linked testing? 12. Are there any ways you feel the study could have been improved? 13. Do you believe that index linked testing should be continued after you leave the study clinics? If yes, why? If no, why not? |
| **Experiences with HIV positive participants** |  | 1. In general, how did participants who had children who tested HIV positive through B-GAP initially react? 2. How did they feel about the home visit intervention when you proposed it? 3. What were your experiences offering the home visit intervention to caregivers? 4. Where there any differences between participants in urban and rural areas? Can you describe these differences? 5. Were there any differences between participants with older and younger children? 6. Between male and female caregivers? 7. With participants whose children were biological vs. non-biological children? 8. Can you describe your experiences working with B-GAP community health workers? 9. Can you describe some of their strengths? 10. Can you describe some of their weaknesses? 11. From the clinic perspective what are the ways you feel the home visit intervention can be improved? |
| **Open session** |  | 1. Before ending, is there anything else you would like to add that we may not have covered in this discussion? |
